# Long Noncoding RNA *NTT* Context-Dependently Regulates *MYB* by Interacting With Activated Complex in Hepatocellular Carcinoma Cells

**DOI:** 10.3389/fonc.2021.592045

**Published:** 2021-09-20

**Authors:** Ya-Sian Chang, Ya-Ting Lee, Ju-Chen Yen, Yuli C. Chang, Li-Li Lin, Wen-Ling Chan, Wei-Chiao Chang, Shyr-Yi Lin, Jan-Gowth Chang

**Affiliations:** ^1^Epigenome Research Center, China Medical University Hospital, Taichung, Taiwan; ^2^Center for Precision Medicine, China Medical University Hospital, Taichung, Taiwan; ^3^School of Medicine, China Medical University, Taichung, Taiwan; ^4^Department of Medical Research, Kaohsiung Medical University Hospital, Kaohsiung, Taiwan; ^5^Department of Bioinformatics and Medical Engineering, Asia University, Taichung, Taiwan; ^6^Department of Clinical Pharmacy, School of Pharmacy, College of Pharmacy, Taipei Medical University, Taipei, Taiwan; ^7^Division of Gastroenterology, Department of Internal Medicine, Wan Fang Hospital, Taipei Medical University, Taipei, Taiwan; ^8^Department of General Medicine, School of Medicine, College of Medicine, Taipei Medical University, Taipei, Taiwan

**Keywords:** long noncoding RNA, *NTT*, *MYB*, hepatocellular carcinoma, context-dependent

## Abstract

**Background:**

Long noncoding RNA (lncRNA) mediates the pathogenesis of various diseases, including cancer and cardiovascular, infectious, and metabolic diseases. This study examined the role of lncRNA *NTT* in the development and progression of cancer.

**Methods:**

The expression of *NTT* was determined using tissues containing complementary DNA (cDNA) from patients with liver, lung, kidney, oral, and colon cancers. The expression of *cis-*acting genes adjacent to the *NTT* locus (*CTGF*, *STX7*, *MYB*, *BCLAF1*, *IFNGR1*, *TNFAIP3*, and *HIVEP2*) was also assessed. We used knockdown and chromatin immunoprecipitation (ChIP) assays to identify the *cis-*acting genes that interact with *NTT*.

**Results:**

*NTT* was most significantly downregulated in hepatocellular carcinoma (HCC), while a higher *NTT* level correlated with a shorter survival time of patients with HCC. Multivariate analysis indicated *NTT* was not an independent predictor for overall survival. *MYB* was significantly upregulated, and its increased expression was associated with dismal survival in HCC patients, similar to the results for *NTT*. *NTT* knockdown significantly decreased cellular migration. ChIP of HCC cell lines revealed that *NTT* is regulated by the transcription factor ATF3 and binds to the *MYB* promoter *via* the activated complex. Additionally, when *NTT* was knocked down, the expression of *MYB* target genes such as *Bcl-xL*, *cyclinD1*, and *VEGF* was also downregulated. *NTT* could play a positive or negative regulator for *MYB* with a context-dependent manner in both HCC tissues and animal model.

**Conclusion:**

Our study suggests that *NTT* plays a key role in HCC progression *via MYB*-regulated target genes and may serve as a novel therapeutic target.

## Introduction

Hepatocellular carcinoma (HCC) is one of the most common cancers and the third leading cause of cancer-related deaths worldwide (1). HCC accounts for more than 90% of liver cancers (2). Globally, approximately 600,000 patients with HCC die annually, and 78,200 new cases are diagnosed. Its incidence is highest in Asia and Africa and is higher among male than female individuals. The risk factors for developing HCC include infection with hepatitis B or C virus, alcohol abuse, nonalcoholic fatty liver disease, Budd–Chiari syndrome, aflatoxin B1 intake, and metabolic diseases (3, 4). Serum alpha-fetoprotein (AFP) and ultrasonography are used clinically for the early detection of HCC (5). However, the sensitivity and specificity of AFP for the diagnosis of HCC (cutoff 20 ng/ml) are 53% and 90%, respectively. Therefore, AFP has been removed from the HCC surveillance guidelines of the American Association for the Study of Liver Disease due to its low diagnostic accuracy (2, 6, 7). Cell-free nucleic acids could also contribute to the surveillance and diagnosis of HCC (8). Furthermore, because of the high postoperative tumor recurrence rate, which can reach 50%, surgical resection is effective for only about 20% of HCC patients (9). The overall 5-year survival is as low as 11–30% (10). A better understanding of the molecular mechanisms underlying the progression of HCC may contribute to the identification of therapeutic targets or diagnostic and prognostic markers.

Long noncoding RNAs (lncRNAs) are noncoding RNAs more than 200 nucleotides long. Because the expression of lncRNAs is lower than that of messenger RNAs (mRNAs), lncRNAs were considered transcriptional noise. However, lncRNAs play crucial roles in regulating various cellular processes and tumorigenesis, such as cell proliferation, metastasis, differentiation, and genomic instability *via* transcription, posttranscription, and epigenetic modifications of the related gene expression (11–15). Four typical molecular functions of lncRNAs are signal, decoy, manipulation, and scaffolding (16). The lncRNAs *H19*, *HOTAIR*, *HULC*, *MALAT1*, *NEAT1*, *TUG1*, *UCA1*, and *ZFAS1* have been reported to be associated with HCC initiation, progression, and metastasis in humans (17).

Noncoding transcript in T cells (*NTT*) is located on chromosome 6q23-q24 and transcribes a 17-kb polyadenylated RNA, which is not spliced. It was first described in 1997 in activated CD4+ T cells (18). Subsequently, Amarante et al. detected *NTT* in peripheral blood mononuclear cells following stimulation with human immunodeficiency virus (HIV) peptide, suggesting it has a role in cellular immune responses (19). A recent study demonstrated that the *NTT/PBOV1* axis promotes monocyte differentiation and is elevated in rheumatoid arthritis (20). *NTT* expression in myalgic encephalomyelitis/chronic fatigue syndrome (ME/CFS) has also been investigated (21). However, the expression pattern, biological function, and underlying mechanism of *NTT* in HCC are still unclear.

In this study, we analyzed *NTT* levels in HCC tissues and their correlation with the clinicopathological characteristics and prognosis of patients with HCC. Mechanistically, we suggest that lncRNA *NTT* interacts with *MYB* and epigenetically activates downstream target genes to facilitate cell migration in HCC. *In vivo* and *in vitro* assays indicated that *NTT* context-dependently regulated *MYB*.

## Materials and Methods

### Patient Samples

The samples examined in this study were from liver (n = 80), lung (n = 36), kidney (n = 34), oral (n = 28), and colon (n = 15) cancers. Tumor and adjacent normal tissues were collected from patients who underwent surgery at China Medical University Hospital. All samples were immediately snap-frozen in liquid nitrogen and stored at −80°C. The study was approved by the Research Ethics Committee of China Medical University Hospital (CMUH102-REC1-037).

### RNA Extraction, Reverse Transcription, and Real-Time Quantitative Polymerase Chain Reaction

Total RNA was isolated using a NucleoSpin^®^ RNA Kit (Macherey Nagel, Düren, Germany) and reverse transcribed into complementary DNA (cDNA) using a high-capacity cDNA Reverse Transcription Kit (Applied Biosystems, Foster City, CA, USA). Quantitative PCR (qPCR) was performed using the IQ2 TaqMan Probe System (Biogenesis, Taiwan) on a LightCycler 480 instrument (Roche Diagnostics, Mannheim, Germany) in accordance with the manufacturer’s instructions. The 2^−ΔΔCt^ method was used to calculate the relative gene expression normalized to glyceraldehyde 3-phosphate dehydrogenase (GAPDH). Primer and probe sequences are listed in **Supplementary Table 1**.

### Protein Extracts and Western Blotting

Proteins were extracted using a cell lysis solution [20 mM Tris/HCl, pH 7.5, 150 mM NaCl, 1 mM ethylenediaminetetraacetic acid (EDTA), 1% Nonidet P-40, 1% sodium deoxycholate, 2.5 mM sodium pyrophosphate, 1 mM b-glycerophosphate, 1 mM Na_3_VO_4_, and 1 μg·ml^−1^ leupeptin] and separated by sodium dodecyl sulfate/polyacrylamide gel electrophoresis (SDS/PAGE). After proteins were transferred onto polyvinylidene difluoride membranes (Millipore), the membranes were blocked with 5% bovine serum albumin (BSA) (Santa Cruz Biotechnology) and then exposed at 4°C overnight to the anti-c-MYB (Abcam, Cambridge, MA, USA) and anti-GAPDH (GeneTex, Inc., Taiwan), followed by horseradish peroxidase-conjugated secondary antibody for detection by an ECL chemiluminescence detection system (GE Healthcare, Pittsburgh, PA, USA).

### Cell Culture and shRNA Transfection

The HCC cell lines (Huh7 and HepG2) were maintained in our laboratory and cultured in Dulbecco’s modified Eagle’s medium (GIBCO BRL, Gaithersburg, MD, USA) supplemented with 10% fetal bovine serum (GIBCO BRL), 100 U/ml penicillin, and 100 mg/ml streptomycin in a humidified incubator containing 5% CO_2_ at 37°C. We used short tandem repeats to authenticate HCC cell lines that were used in this study. Primary human hepatocytes (HH) was from nearby normal parts of HCC tissues used as normal liver control.

For *NTT* knockdown, short hairpin RNA (shRNA) (5′-CAGAGCTGACTACACGGAGTGTT-3′) and negative control shRNA (5′-TTCTCCGAACGTGTCACGTTT-3′) were synthesized by MDBio (Taipei, Taiwan). The *NTT* and negative control shRNAs were constructed in pSUPER.neo (OligoEngine, Seattle, WA, USA). Huh7 and HepG2 cells were transiently transfected with shRNA or negative control using Lipofectamine 2000 (Invitrogen, Carlsbad, CA, USA), and a stable clone was selected by G418. The transfection efficiency was determined by qPCR.

For *ATF3* knockdown, sh*ATF3* was obtained from the National RNAi Core Facility at Academia Sinica (Taiwan). The shRNA sequence was used for *ATF3* knockdown (5′-GCTGAACTGAAGGCTCAGATT-3′).

### Cytoplasmic and Nuclear RNA Extraction

Cytoplasmic and nuclear cell lysates were isolated using the NE-PER Nuclear and Cytoplasmic Extraction Kit (Thermo Fisher Scientific, Waltham, MA, USA) according to the manufacturer’s instructions followed by TRIzol RNA extraction. The nuclear fraction was determined by qPCR.

### Wound-Healing Assay

A wound-healing assay was used to assess cell migration. Huh7 and HepG2 cells were seeded and grown in 24-well plates until a monolayer formed. The cells were then scratched using a 200-μl plastic tip to create a straight wound. Wound gaps were photographed and analyzed using the free TScratch software.

### Cell Proliferation Assay

Cell proliferation was assessed using a trypan blue exclusion assay. Cells were plated in 96-well plates at 1 × 10^5^ cells per well and incubated for 24 or 48 h. After incubation, the number of cells was determined using the 3-(4,5-dimethylthiazol-2-yl)-2,5-diphenyltetrazolium bromide (MTT) assay.

### RNA-Binding Protein Immunoprecipitation

RNA-binding protein immunoprecipitation (RIP) was conducted using the RNA ChIP-IT kit (Active Motif, Carlsbad, CA, USA) according to the manufacturer’s instructions. Briefly, after cross-linking, cells were lysed, and the lysates were incubated with protein G agarose beads conjugated with specific antibodies (anti-EZH2, anti-EED, anti-MLL1, anti-PML, or anti-H3H4me3) or control immunoglobulin G (IgG). Then, the beads were incubated with proteinase K, and the purified RNA was subjected to qRT-PCR.

### DNA ChIP

DNA ChIP was performed using the ChIP-IT kit (Active Motif). Cells were treated with formaldehyde and incubated for 10 min to generate DNA protein cross-links. The cell lysates were sonicated to obtain 200–500-bp chromatin fragments and immunoprecipitated with a specific antibody (anti-PML, anti-H3K4me3, anti-EZH2, or anti-ATF3) or IgG as the control. Precipitated chromatin DNA was recovered and analyzed by qPCR. The *MYB* promoter primer sequences were 5′-CCTAGCCAAACAGCCTATGAA-3′ (forward) and 5′-TGGAGACGGGGAAATTAGG-3′ (reverse). The *NTT* promoter primer sequences were 5′-CACCCACATGGTAGACAGGA-3′ (position 5350 forward) and 5′-CCCAGCTCCCAGAAGATACA-3′ (reverse).

### Fluorescent *In Situ* Hybridization

Cells were harvested by directly culturing on cover slips. After fixation in 1% formaldehyde, the slides were inserted into solution A [80% formamide, 10% 1× saline sodium citrate (SSC) and 10% H_2_O] at room temperature for 2 min. Then, they were quenched in ice-cold 70% ethanol for 5 min to fix the slides. The RNA fluorescence *in situ* hybridization (FISH) locked nucleic acid (LNA) probe (corresponding to the sequence of U54776.1 from 10,024 to 10,044) was purchased from Exiqon Inc. (MA, USA) and labeled in orange. The slides were dehydrated, air dried, and preheated to 37°C for 2 min before probe addition. The slides were incubated overnight at 37°C in a humid chamber in an incubator. After RNA FISH washing, the slides were postfixed prior to DNA FISH in 4% paraformaldehyde for 15 min at room temperature and rinsed once with phosphate-buffered saline (PBS). The slides were then denatured in 70% formamide and 2× SSC at 80°C for 10 min. The slides were dehydrated before the denatured probes were added for overnight hybridization at 42°C in a dark humid environment. DNA FISH probe were purchased from Cytocell (Cat. No. LPH016). The red 183-kb *MYB* probe covers the entire *MYB* gene; the probe mix also contains a green control probe for the chromosome 6 centromere probe (*D6Z1*). Then, the slides were washed twice in 2× SSC at 45°C, and 4′,6-diamidino-2-phenylindole (DAPI) was added to this slides. Images were obtained using an E600 microscope (Nikon, Tokyo, Japan) with CytoVision software (Applied Imaging, Santa Clara, CA, USA).

Formalin-fixed, paraffin-embedded HCC and paired non-tumor tissue sections (1–2 μm) on poly-l-lysine-coated slides were deparaffinized. Before hybridization, the slides were incubated with 10% pepsin for 30 min at 37°C and then heated in denaturation solution at 72°C for 3 min. The RNA FISH probe (corresponding to the sequence of U54776.1 from 13,559 to 13,620) was labeled with Cy3 dyes using the Label IT labeling kit (Mirus, Madison, WI, USA). Hybridization with the denatured *NTT* probe was performed in a humid chamber at 37°C overnight. Then, the slides were washed at 45°C in washing buffer for 5 min. Finally, the slides were counterstained with DAPI and analyzed by fluorescence microscopy.

### Animal Experiments

BALB/c athymic nude mice (male, 4–6 weeks old) were purchased from the National Laboratory Animal Breeding and Research Center, Taiwan. To establish a HCC xenograft model, 1 × 10^7^ scramble or sh*NTT*-Huh7 cells were suspended in 100 ml PBS and inoculated subcutaneously into the flanks of eight nude mice (left: sh*NTT*; right: scramble). All animal experiments were performed in accordance with the guidelines set by the Institutional Animal Care and Use Committee (IACUC) of China Medical University (CMU). All animals were housed in the Laboratory Animal Center of CMU under a 12 h light/dark (08:00/20:00) cycle with free access to food and water. The mice were sacrificed using CO_2_, and the tissues were subsequently harvested. All breeding and subsequent use of animals in this study, including sacrifice, was approved by the IACUC of CMU. The IACUC approval number was 102-203-N.

### Luciferase Reporter Assay

Fragments of DNA from the *NTT* promoter region were obtained through PCR amplification and cloned into the pGL3-basic reporter vector (Promega Corporation). The constructs were transfected into Huh7 cells (1 × 10^5^) using Lipofectamine 2000 (Thermo Fisher Scientific, Inc.). The cells were lysed and assayed for luciferase activity using Steady-Glo Luciferase assay system (Promega Corporation) according to the manufacturer’s protocol at 24 h posttransfection.

### Statistical Analysis

Student’s *t*-test was used to test for differences in mean values between two groups. Correlations between the *NTT* level and clinicopathological characteristics of the HCC patients were analyzed with the chi-square test. Survival analysis was performed using the Kaplan–Meier method, followed by the log-rank test. The level of statistical significance was set at *p* < 0.05. All statistical analyses were performed using GraphPad Prism software (ver. 8.0.2; GraphPad Software, Inc., La Jolla, CA, USA).

## Results

### *NTT* Expression in Various Cancer Types

**Figure 1:**
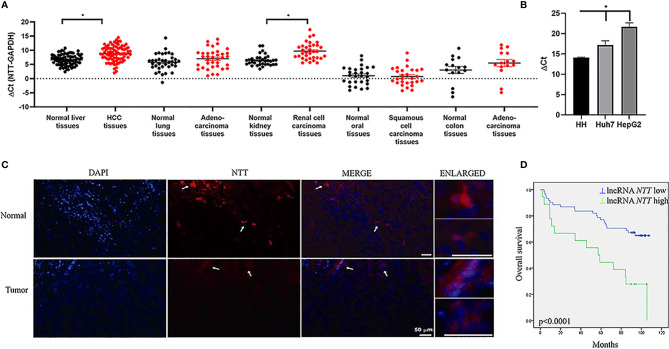
*NTT* is dysregulated in various cancers. **(A)** Expression of *NTT* was evaluated by qPCR in different cancer types. **(B)** Low expression of *NTT* in HCC cell lines, including Huh7 and HepG2 in comparison with that in normal primary human hepatocytes. **(C)** *NTT* expression was also evaluated in HCC by RNA FISH. **(D)** High *NTT* expression indicated a poor clinical outcome. *p < 0.05.

We explored *NTT* expression in liver (HCC), lung (adenocarcinoma), kidney (renal cell carcinoma), oral (squamous cell carcinoma), and colon (adenocarcinoma) cancers. *NTT* expression was higher in cancer than in normal tissue in oral cancer but lower in cancer compared with normal tissues in the other types of cancer (**Figure 1A**). Our data showed that *NTT* was dysregulated in cancers. *NTT* showed the highest differences in HCC compared to its surrounding normal tissue, in respect to lung, kidney, oral, and colon cancers. Next, we investigated *NTT* expression in normal hepatocytes and HCC cell lines (Huh7 and HepG2). We found that the *NTT* expression in normal hepatocytes was higher than in Huh7 and HepG2 (**Figure 1B**), similar to the findings for HCC and nearby tissues. FISH confirmed that *NTT* expression was lower in tumor cells than in non-tumor cells (**Figure 1C**). Based on these results, we focused on *NTT* in HCC and the HCC cell lines Huh7 and HepG2 in the following experiments.

### The *NTT* Level Is Correlated With the Clinical Characteristics of Patients With HCC

Using the mean *NTT* expression level in the 80 HCC patients, 62 patients (77.5%) were classified into a low-*NTT* expression group, and the remaining 18 patients (22.5%) were classified into a high-*NTT* expression group. Further survival analysis revealed that higher *NTT* expression was correlated with a shorter survival time in patients with HCC (*p* < 0.0001, **Figure 1D**). Moreover, there were significant correlations between *NTT* expression and age, tumor size, and tumor stage (**Supplementary Table 2**). In the multivariate analysis, which incorporated independent prognostic factors of gender, age, differentiation, tumor size, and stage, we found that *NTT* was not an independent predictor for overall survival (*p* = 0.059) (**Supplementary Table 3**).

### Expression of Genes Near *NTT* in HCC: *CTGF*, *STX7*, *MYB*, *BCLAF1*, *IFNGR1*, *TNFAIP3*, and *HIVEP2*

**Figure 2:**
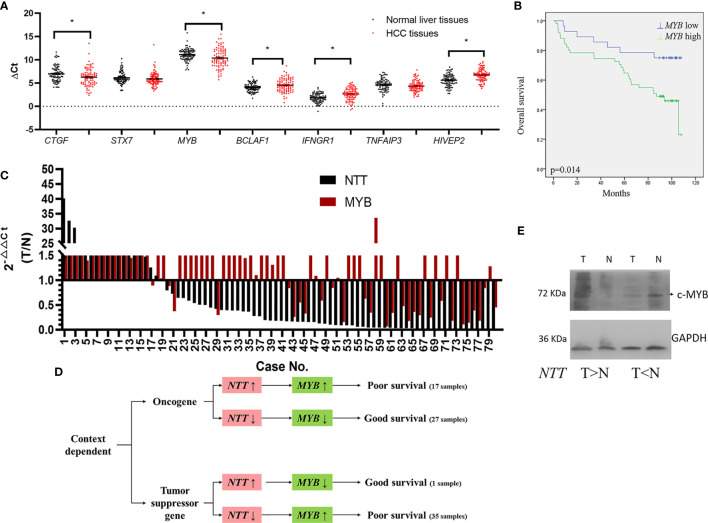
Genes near *NTT* are dysregulated in HCC. **(A)** Expression of *CTGF*, *STX7*, *MYB*, *BCLAF1*, *IFNGR1*, *TNFAIP3*, and *HIVEP2* was evaluated by qPCR in HCC. **(B)** High *MYB* expression indicated a poor clinical outcome. *p < 0.05. **(C)** Log2 fold changes in *NTT* and *MYB* expressions (T/N) of each case are plotted. **(D)** The roles of *NTT* for *MYB* is context dependent. **(E)** *MYB* protein expression in HCC tissue samples. Left pair was *MYB* increased in HCC; right pair was *MYB* decreased in HCC.

lncRNA usually plays an important role in the regulation of genes nearby. We measured the expression of genes near *NTT* in HCC tissues. The genes were as follows: *CTGF*, *STX7*, *MYB*, *BCLAF1*, *IFNGR1*, *TNFAIP3*, and *HIVEP2* (**Supplementary Figure 1**). The expression of *CTGF* and *MYB* was significantly higher in HCC tissues than that in the adjacent non-tumor tissues (*p* < 0.05, **Figure 2A**), while the expression of *BCLAF1*, *IFNGR1*, and *HIVEP2* was significantly lower in HCC tissues than in adjacent non-tumor tissues (*p* < 0.05, **Figure 2A**). The high *MYB* levels in tumors predicted a poor clinical outcome (*p* = 0.014, **Figure 2B**), similar to the results for *NTT* expression. Based on these results, we explored the relationship between *MYB* and *NTT*, and the regulation mechanism.

First, we analyzed the correlation between the expressions of *NTT* and *MYB* in human tissues (**Figure 2C**). We found that *NTT*-positive regulation of *MYB* expression in 55% (44/80) of the HCC tissues, which including both *NTT* and *MYB* expressions were increased in 17 HCC tissues and both expressions were decreased in 27 HCC tissues (**Figure 2D**). From these results, we suggested that *NTT* plays like an oncogene.

However, *NTT* negative regulation of *MYB* expression in 45% (36/80) of the HCC tissues, which contained *NTT* expression, was decreased, and *MYB* expression was increased in 35 HCC tissues; *NTT* expression was increased and *MYB* expression was decreased in one HCC tissue (**Figure 2D**).

No differences were observed in the survival rates between patients with *NTT* positive and negative regulation of *MYB* expression (*p* = 0.796) (**Supplementary Figure 2A**). Further survival analysis revealed that there was significant correlation between higher and lower expression of both *NTT* and *MYB* (*p* = 0.000) (**Supplementary Figure 2B**). Survival is lower among both *NTT-* and *MYB*-upregulated patients compared with both *NTT-* and *MYB*-downregulated patients (**Supplementary Figures 2B, D** **)**.

We also found that *MYB* play a determining role for HCC survival, overexpression of *MYB* resulted in poor survival, and downexpression of *MYB* correlated with good survival (**Figure 2D**). The expression of *NTT* could not determine patient’s survival directly. These data support the context-dependent role of *NTT* in liver tumorigenesis. Western bot analysis was also done to confirm the MYB protein expression in HCC tissue samples, the results of representative cases showed that *NTT* overexpression upregulated the expression of MYB, and *NTT* downexpression downregulated the expression of MYB in the paired normal and tumor tissues (**Figure 2E**).

### *NTT* Promotes Cell Migration in HCC Cell Lines

**Figure 3:**
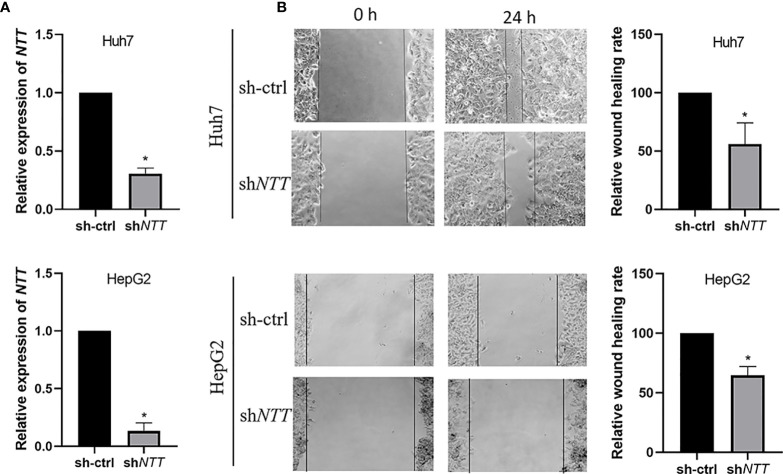
*NTT* knockdown reduced cell migration ability. **(A)** Expression of *NTT* was determined by qPCR. **(B)** Knockdown of *NTT* inhibited cell migration. Data are shown as the mean ± standard deviation (n = 3). *p < 0.05 for sh-ctrl *vs.* sh*NTT*.

We further explored the biological role of *NTT* in HCC cell lines. The sh*NTT* plasmid was transfected into HCC Huh7 and HepG2 cells, which effectively knocked down the expression of *NTT* (**Figure 3A**). Knockdown of *NTT* significantly reduced the number of migratory cells in Huh7 and HepG2 cells compared with the sh-ctrl transfected cells (**Figure 3B**).

To test whether *NTT* levels affect cell proliferation, viable cells were counted using the MTT assay after Huh7 cells were transfected with shRNA for 48 h. We found no difference in cell proliferation between the Huh7 cells transiently transfected with sh-ctrl and sh*NTT* (**Supplementary Figure 3**).

### *NTT* Regulates the Nearby *MYB* Gene *via* Activated Complex Binding

**Figure 4:**
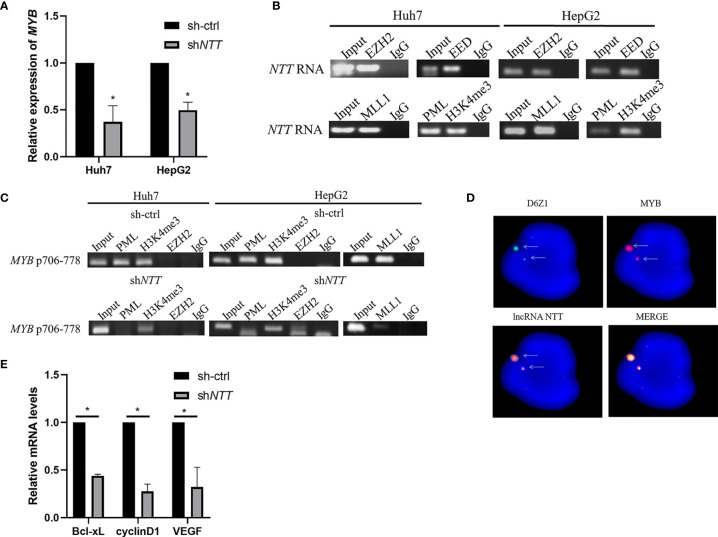
*MYB* expression may be regulated by *NTT* through its interaction with the activated complex. **(A)** *MYB* expression was examined in Huh7 and HepG2 cells transfected with *NTT* shRNA. **(B)** RIP-RT-qPCR analysis of *NTT* RNA binding to PRC2 (EZH2 and EED) and activated complex (MLL1, PML, and H3K4me3). **(C)** DNA ChIP-qPCR analysis of PML and H3K4me3 occupancy of the *MYB* promoter region. **(D)** RNA-DNA FISH conforming that *NTT* interacts with *MYB*. **(E)** RT-qPCR was used to measure expression of the selected genes in *NTT* knockdown cells. *p < 0.05.

Next, we investigated how *NTT* regulates the *MYB* gene. *NTT* expression in Huh7 and HepG2 were knocked down by shRNA, and the relative *MYB* mRNA level was analyzed (**Figure 4A**). Transfection of sh*NTT* in Huh7 and HepG2 led to decreased *MYB* expression.

Since it has been reported that lncRNAs may bind to polycomb-repressive complex 2 (PRC2), which consists of EZH2, EED, and SUZ12, to repress the expression of downstream genes, we examined whether *NTT* binds to the *MYB* promoter *via* interaction with PRC2. An RIP assay showed that *NTT* could bind EZH2 and EED (**Figure 4B**). Furthermore, we found that *NTT* not only bound to PRC2 but also bound to the activated complex (MLL1, PML, and H3K4me3) to activate gene transcription (**Figure 4B**). A similar result was observed with the HepG2 cells (**Figure 4B**).

DNA ChIP showed that the activated complex binds to positions 706–778 of the *MYB* promoter, and activated complex binding became undetectable or reduced following *NTT* knockdown (**Figure 4C**), suggesting that *NTT* enhances *MYB* expression by interacting with the activated complex binding to the *MYB* promoter. In agreement with the above results, we also found that *NTT* knockdown reduced the binding of PML and MLL1 to the *MYB* promoter (**Figure 4C**).

To further confirm the localization of *NTT* RNA transcript with *MYB* gene area, we used DNA (*MYB* genomic probe) FISH to detect the genomic location of *MYB* and RNA (*NTT* 21 base pair LNA probe) FISH to detect the *NTT* RNA transcript. Colocalization of *NTT* transcripts with the *MYB* gene was revealed (**Figure 4D**). These results suggest that *NTT* modulates nearby genes, such as *MYB*, *via* RNA transcripts and related protein complexes.

To explore the molecular mechanism underlying MYB-induced tumor promotion and metastasis, genes associated with tumor progression and metastasis were examined in sh-ctrl and sh*NTT* cells by real-time quantitative PCR (RT-qPCR). *Bcl-xL*, *cyclinD1*, and *VEGF* are associated with cell proliferation, the cell cycle, and cell migration/invasion, respectively. All three were downregulated following *NTT* knockdown (**Figure 4E**).

### *NTT* Is Regulated by ATF3

**Figure 5:**
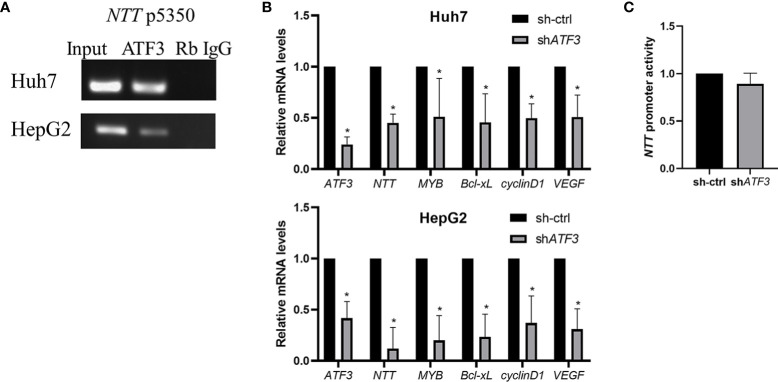
The functional effects of *NTT* are modulated by ATF3. **(A)** DNA ChIP-qPCR analysis of ATF3 occupancy of the *NTT* promoter region, shown by a representative graph of three independent experiments. **(B)** Knockdown of *ATF3* by shRNA resulted in lower expression of *NTT* and its downstream genes. *p < 0.05. **(C)** Activity of promoter determined using a luciferase assay upstream of *NTT*.

Examining the *NTT* promoter sequence, we identified the potential binding motif of ATF3, a key transcription factor in HCC cell lines. To verify if ATF3 binds to the *NTT* promoter and regulates *NTT* expression, we performed DNA ChIP and shRNA knockdown of *ATF3*. ATF3 binding was detected at position 5350 of the *NTT* promoter in Huh7 and HepG2 (**Figure 5A**), and *ATF3* knockdown resulted in decreased *NTT* and its downstream genes expression (**Figure 5B**).

We have performed the luciferase reporter assays to determine the *NTT* promoter activity in *ATF3*-knockdown cells, and the results showed that the luciferase activity was decreased after knockdown *ATF3* (**Figure 5C**).

### *NTT* Context-Dependently Regulating *MYB In Vivo*

We constructed stable cell lines that knocked down *NTT* or sh-ctrl control to verify the effect of *NTT* on tumorigenicity. From the *in vivo* experiments, we could not define *NTT* is an oncogene or tumor suppressor. Therefore, we examined *NTT* and *MYB* expression of eight mice tumor samples. The results showed both *NTT* and *MYB* overexpression result in larger tumor size than control in three-fourths of the mice, and *NTT* downexpression with *MYB* overexpression may result in either larger or smaller tumor or no change size than control (one-third with small, one-third with larger, and one-third with no definite change than control), and one mouse with both downexpression of *NTT* and *MYB* showed slight size change than the control (**Supplementary Figure 4**). These data are consistent with the findings of the human tissues experiments and suggested a context-dependent function of *NTT* for the regulation of *MYB* expression in in HCC development.

## Discussion

Many studies have reported that lncRNAs are involved in the regulation of HCC differentiation and cell growth, including cell proliferation and apoptosis, invasion, and metastasis. For example, the lncRNA *HOTAIR* promotes cell invasion, proliferation, and metastasis and activates autophagy (22). *HOTAIR* also mediates hepatocarcinogenesis by suppressing *miRNA-218* expression and activating P14 and P16 signaling (23). The lncRNA *MALAT1* is associated with cell proliferation and migration by regulating Bax, bcl-2, bcl-xl, caspase-3, and caspase-8 (24). *HULC* promotes cell proliferation and triggers autophagy (25, 26). *MEG3* expression in HCC samples is downregulated compared with normal controls. *MEG3* interacts with the p53 DNA binding domain directly and regulates partial p53 target genes in hepatoma cells (27). As the role of *NTT* in HCC tumorigenesis is unknown, we explored it herein.

*NTT* was identified during the activation of T cells with phytohemagglutinin or with phorbol 12-myristate 13-acetate and ionomycin (18). *NTT* can be induced by synthetic peptide p9 of HIV-1 in human lymphocytes (19). Delgado et al. demonstrated that *NTT* plays a key role in the activation of lymphocytes mediated by RNA-dependent protein kinase through nuclear factor-kappa B activation (28). Recent studies have linked *NTT* overexpression to chronic inflammatory autoimmune disease, rheumatoid arthritis, and ME/CFS (20, 21). However, we did not find any correlations between *NTT* and hepatitis B or C infection in HCC patients. In addition, 17 primary/5 relapse HCC tumor samples and 22 adjacent normal liver tissue samples obtained from Gene Expression Omnibus (GEO) (GSE101432) were included in this study to validate the role of *NTT* in HCC. *NTT* is declined in HCC tissues compared with normal liver tissues (**Supplementary Figure 5A**). *NTT* in TCGA-HCC was also identified as compared to normal tissue samples, and decreased expression of *NTT* was also observed in HCC tissues (**Supplementary Figure 5B**). These results were consistent with our findings.

*MYB* is a transcription factor with three functional domains: DNA binding, transactivation, and negative regulatory domains (29). *MYB* is frequently overexpressed in human leukemias, breast cancers, and other solid tumors and is considered an oncogene. *MYB* overexpression promoted cell growth, cell-cycle progression, survival, and malignant behavior. Moreover, *MYB* is thought to be a potential therapeutic target in leukemia and β-hemoglobinopathies (30, 31). MYB has also been shown to interact with p300 protein (32). There are reports that *MYB* mRNA expression is higher in HCC tissues than in matched non-tumor tissues, and survival analysis revealed that strong *MYB* expression had lower disease-specific survival rates than in patients with negative *MYB* expression (33–35). Many studies have shown that *MYB* knockdown results in decreased cell migration (34, 36), similar to our results regarding *NTT* knockdown. We suggest that knockdown of either or both *NTT* and *MYB* produces similar results.

In this study, we confirmed that high *MYB* expression, determined by qRT-PCR, was associated with worse overall survival in a cohort of 80 patients with HCC. *miR-424* plays a critical role in HCC tumorigenesis by targeting *MYB* mRNA (37). Liver cancer-associated lncRNAs participate in HCC processes by binding to the regulatory area of oncogenes, which is different from the translational regulation of microRNA (miRNA) (16). Our data suggest that the ability of *NTT* to target *MYB* may be one mechanism of the posttranscriptional control of *MYB*.

In conclusion, we found that *NTT* play a tumor-suppressor and tumor-promoting roles in human HCC tissues mostly from the ability to negatively or positively control *MYB* oncogene and suggested a context-dependent function of *NTT* in tumor development. Moreover, we report for the first time that the oncogenic activity of *NTT* is attributable to its activation of *MYB* by interacting with the activated complex in cellular experiments. Our results suggest that *NTT* may be a novel therapeutic target for the treatment of HCC.

## Data Availability Statement

The original contributions presented in the study are included in the article/**Supplementary Material**. Further inquiries can be directed to the corresponding authors.

## Ethics Statement

The studies involving human participants were reviewed and approved by China Medical University Hospital (CMUH102-REC1-037). The patients/participants provided their written informed consent to participate in this study. All animal experiments were performed in accordance with the guidelines set by the Institutional Animal Care and Use Committee (IACUC) of China Medical University (CMU).

## Author Contributions

Y-SC, Y-TL, and J-CY: investigation, methodology, and writing—original draft preparation. YC and L-LL: conceptualization and data curation. W-LC and W-CC: visualization, software, and supervision. S-YL and J-GC: writing—reviewing and editing. All authors contributed to the article and approved the submitted version.

## Funding

This study was supported by the China Medical University (CMU103-BC-7) and China Medical University Hospital (DMR-110-238).

## Conflict of Interest

The authors declare that the research was conducted in the absence of any commercial or financial relationships that could be construed as a potential conflict of interest.

## Publisher’s Note

All claims expressed in this article are solely those of the authors and do not necessarily represent those of their affiliated organizations, or those of the publisher, the editors and the reviewers. Any product that may be evaluated in this article, or claim that may be made by its manufacturer, is not guaranteed or endorsed by the publisher.
