# Supplementary material for: Long Noncoding RNA NTT Context-Dependently Regulates MYB by Interacting With Activated Complex in Hepatocellular Carcinoma Cells
Source: Front Oncol. 2021 Sep 20;11:592045. doi: 10.3389/fonc.2021.592045 (PMC8488295; doi:10.3389/fonc.2021.592045)
Supplement: Supplementary file 1 [file DataSheet_1.docx]

**Supplementary Figure 1.** Genes around the *NTT* upstream and downstream on the chr6 location which we selected to further analysis.

**
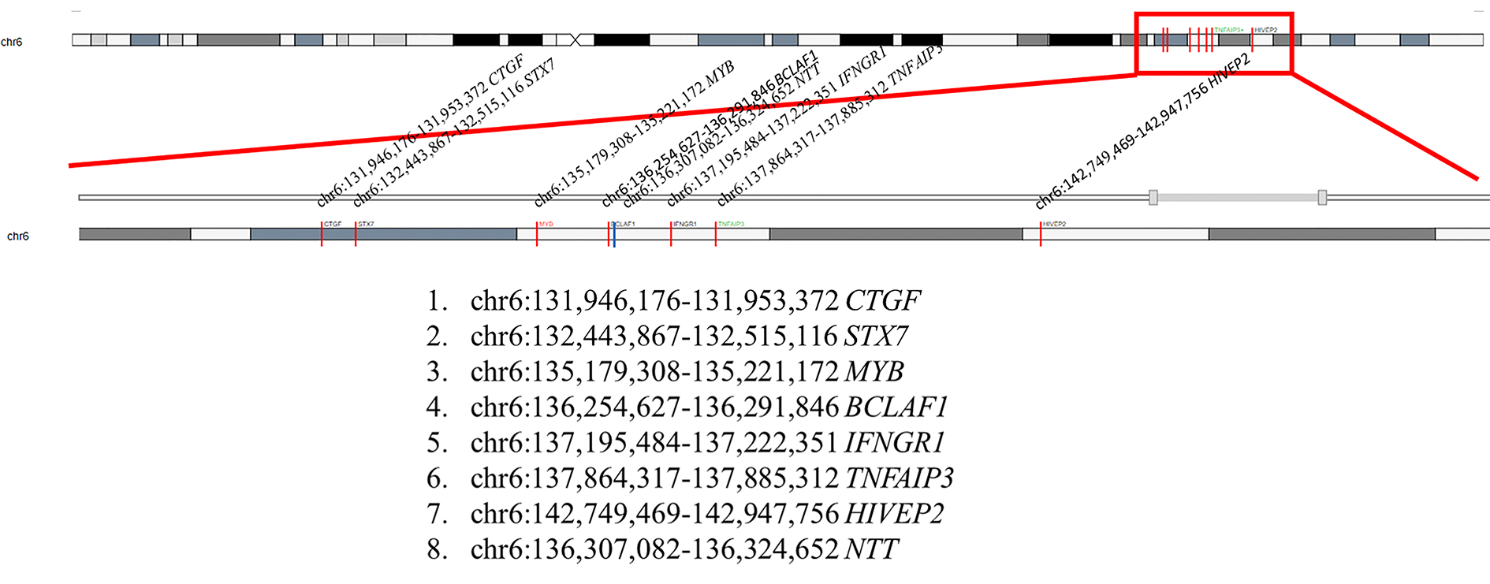
**

**Supplementary Figure 2.** Kaplan-Meier survival curves. (A) *NTT* positive or negative control *MYB* expression (B) Both *NTT* and *MYB* are low or high expression.


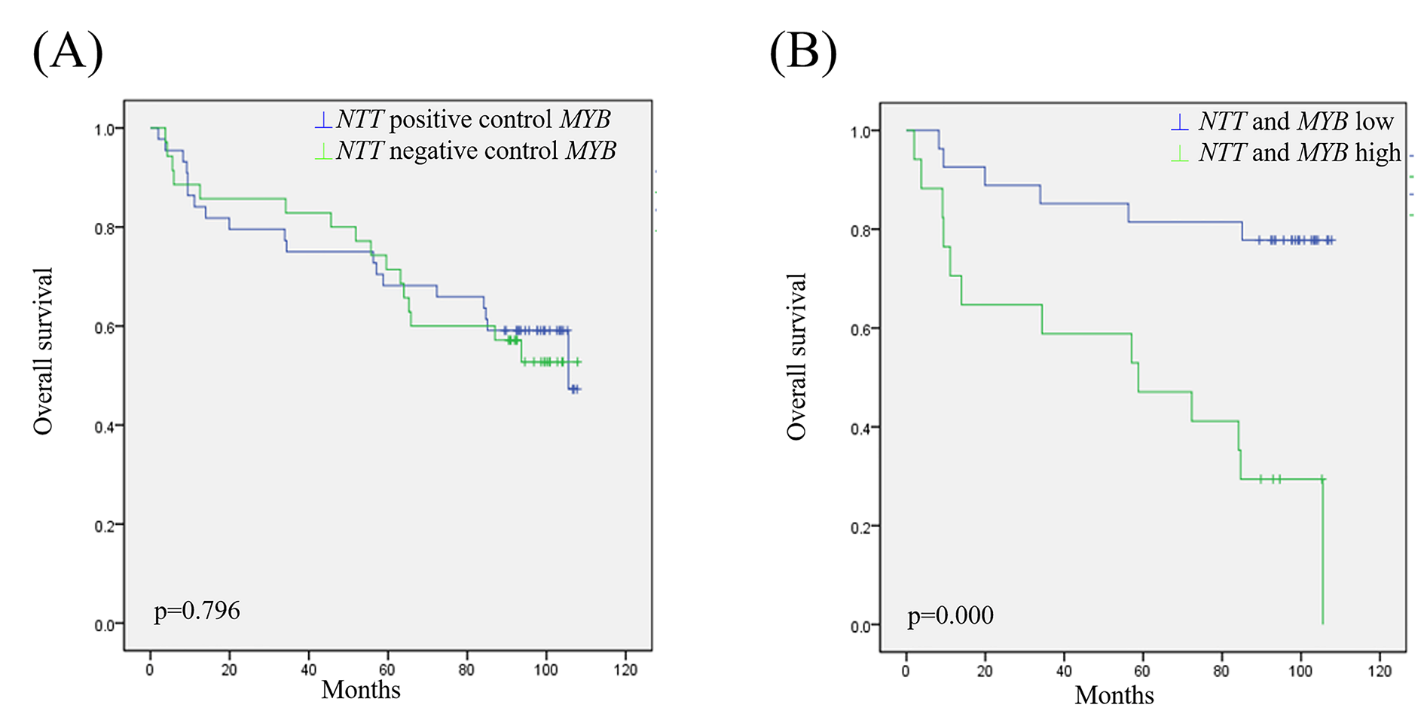


**Supplementary Figure 3.** The effects of *NTT* knockdown on cell growth of Huh7 cells. The cellular proliferation of Huh7 cells transfected with sh-ctrl or sh*NTT*, respecrively, were assessed using MTT assay. Data are shown as the mean ± standard deviation (n = 3). p=0.098 for sh-ctrl *vs*. sh*NTT* showing no statically significance between two groups.


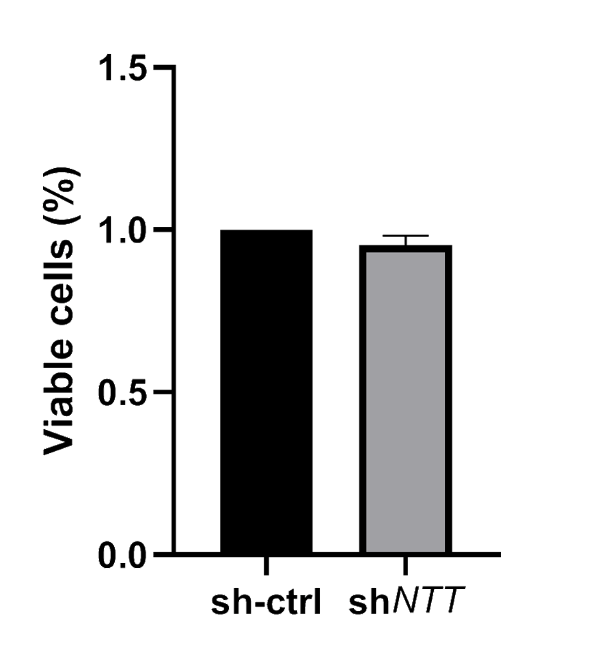


**Supplementary Figure 4.** Mouse tumors in xenograft model analysis.


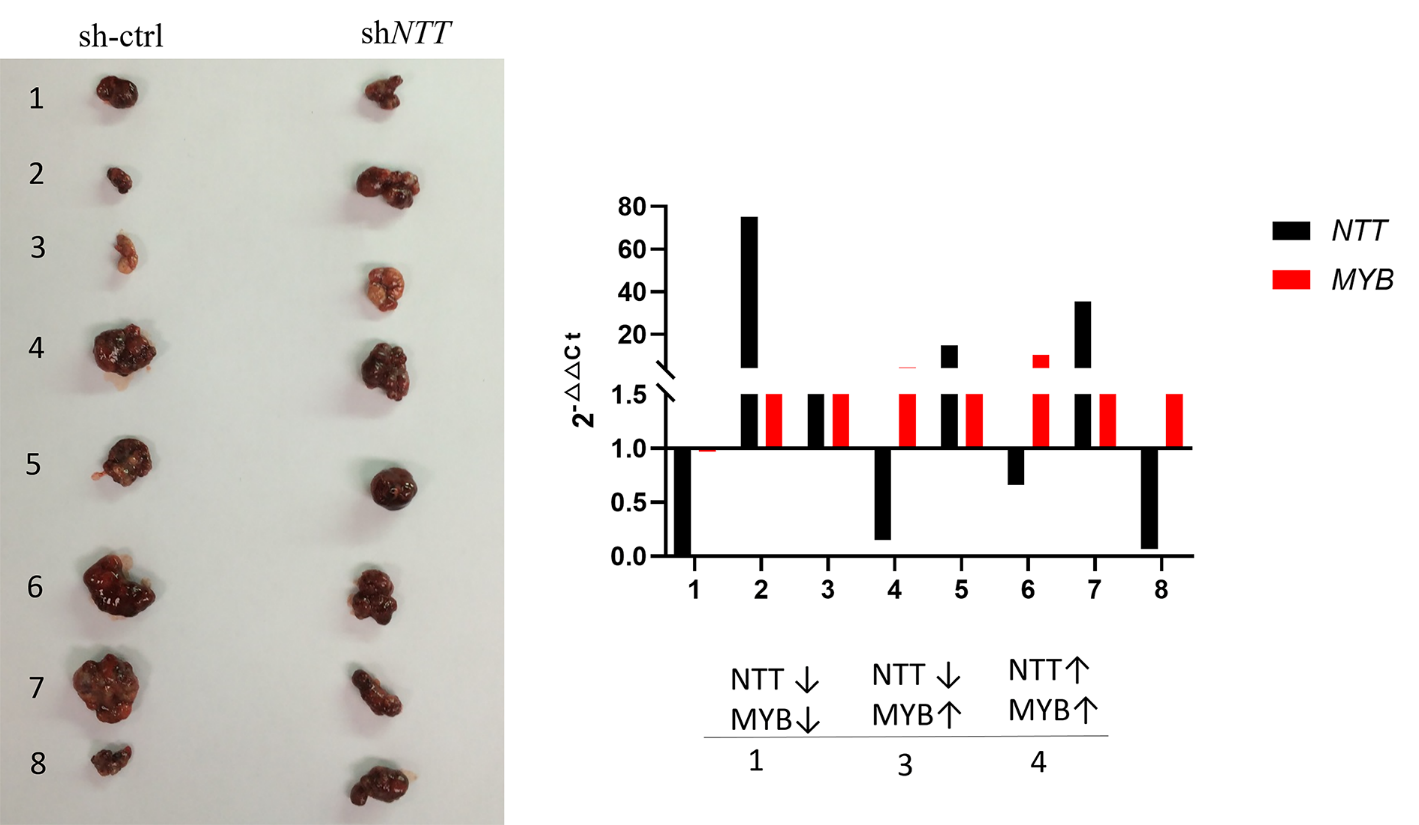


**Supplementary Figure 5.** Low expression of *NTT* in HCC tissues in comparison to that in the non-tumor tissues in (A) GEO dataset (B) TCGA dataset.


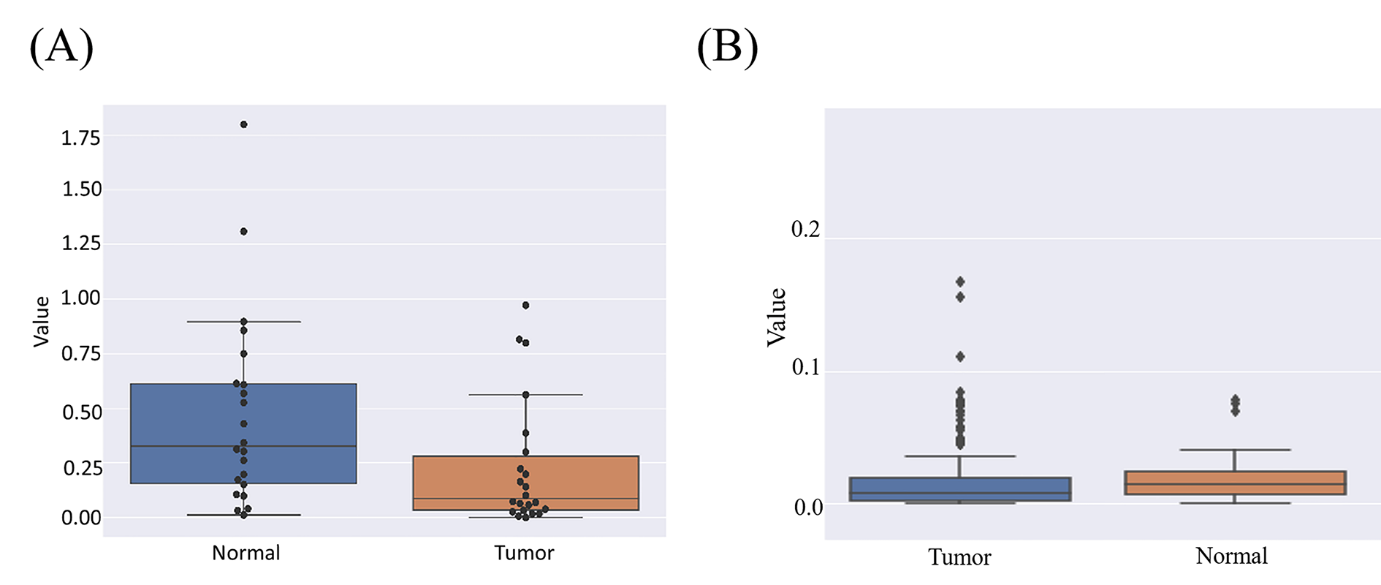


**Supplementary Table 1.** Primer sequences for RT-qPCR

| Gene | Forward primer (5’-3’) | Reverse primer (5’-3’) | Probe (5’-3’) |
| --- | --- | --- | --- |
| *NTT* | cttggcctaaaaggggatg | gcacctttggtctccttcac | #10 |
| *CTGF* | ctcctgcaggctagagaagc | gatgcactttttgcccttctt | #85 |
| *STX7* | accacccccagtgaacag | ggaccttctggaagtttgtca | #53 |
| *MYB* | tgctcctaatgtcaaccgaga | agctgcatgtgtggttctgt | #62 |
| *BCLAF1* | gccgttcctcgttttatcct | actctagactcttcatctgtgaacctt | #22 |
| *IFNGR1* | catgcagggtgtgagcag | aacattagttggtgtaggcactga | #57 |
| *TNFAIP3* | tgcacactgtgtttcatcgag | acgctgtgggactgactttc | #74 |
| *HIVEP2* | cggcaagcttacatcatcaa | aggacgcatcaggtttcatc | #38 |
| *Bcl-xL* | ccttggatccaggagaacg | caggaaccagcggttgaa | #72 |
| *cyclinD1* | ggtagatgtgtaacctcttcacctta | gcacgctacgctactgtaacc | #41 |
| *VEGF* | ctacctccaccatgccaagt | ccatgaacttcaccacttcgt | #63 |
| *ATF3* | tttgccatccagaacaagc | catcttcttcaggggctacct | #87 |
| *GAPDH* | agccacatcgctcagacac | gcccaatacgaccaaatcc | #60 |
| *U2 snRNA* | tttggctaagatcaagtgtagtatctgttc | aatccatttaatatattgtcctcggataga |  |

**Supplementary Table 2.** Correlations between target gene expression and clinicopathological features in HCC.

| Features |  | Cases | *NTT* expression | | *p* value | *MYB* expression | | *p* value | *CTGF* expression | | *p* value | *ATF3* expression | | *p* value |
| --- | --- | --- | --- | --- | --- | --- | --- | --- | --- | --- | --- | --- | --- | --- |
|  |  |  | Low | High |  | Low | High |  | Low | High |  | Low | High |  |
| Gender | Male | 60 | 45 | 15 | 0.354 | 23 | 37 | 0.279 | 20 | 40 | 0.891 | 40 | 20 | 0.117 |
|  | Female | 20 | 17 | 3 |  | 5 | 15 |  | 7 | 13 |  | 17 | 3 |  |
| Age | <60 | 40 | 27 | 13 | 0.032 | 14 | 26 | 1.000 | 14 | 26 | 0.813 | 28 | 12 | 0.805 |
|  | ≥60 | 40 | 35 | 5 |  | 14 | 26 |  | 13 | 27 |  | 29 | 11 |  |
| Differentiation | Low | 34 | 25 | 9 | 0.401 | 8 | 26 | 0.019 | 9 | 25 | 0.401 | 26 | 8 | 0.401 |
|  | Medium | 37 | 31 | 6 | 0.536 | 17 | 20 | 0.136 | 16 | 21 | 0.226 | 26 | 11 | 0.545 |
|  | High | 2 | 2 | 0 |  | 2 | 0 |  | 0 | 2 |  | 1 | 1 |  |
| Size | <4.5 cm | 41 | 37 | 4 | 0.011 | 16 | 25 | 0.604 | 13 | 28 | 0.510 | 30 | 11 | 0.926 |
|  | ≥4.5 cm | 36 | 24 | 12 |  | 12 | 24 |  | 14 | 22 |  | 26 | 10 |  |
| TNM stage | I-II | 55 | 47 | 8 | 0.000 | 24 | 31 | 0.031 | 19 | 36 | 0.287 | 38 | 17 | 0.811 |
|  | III-IV | 11 | 4 | 7 |  | 1 | 10 |  | 2 | 9 |  | 8 | 3 |  |
| Serum AFP | ≤20 ng/mL | 35 | 28 | 7 | 0.379 | 16 | 19 | 0.065 | 11 | 24 | 0.670 | 27 | 8 | 0.053 |
|  | >20 ng/mL | 16 | 11 | 5 |  | 3 | 13 |  | 6 | 10 |  | 8 | 8 |  |
| HBV infection | No | 22 | 17 | 5 | 0.746 | 11 | 11 | 0.084 | 10 | 12 | 0.093 | 15 | 7 | 0.908 |
|  | Yes | 30 | 22 | 8 |  | 8 | 22 |  | 7 | 23 |  | 20 | 10 |  |
| HCV infection | No | 30 | 22 | 8 | 0.746 | 11 | 19 | 0.982 | 12 | 18 | 0.190 | 22 | 8 | 0.279 |
|  | Yes | 22 | 17 | 5 |  | 8 | 14 |  | 5 | 17 |  | 13 | 9 |  |
| Survival | <6 years | 29 | 18 | 11 | 0.015 | 6 | 23 | 0.037 | 8 | 21 | 0.347 | 20 | 9 | 0.775 |
|  | ≥6 years | 50 | 43 | 7 |  | 22 | 28 |  | 19 | 31 |  | 36 | 14 |  |

| Features |  | Cases | *BCLAF1* expression | | *p* value | *IFNGR1* expression | | *p* value | *HIVEP2* expression | | *p* value |
| --- | --- | --- | --- | --- | --- | --- | --- | --- | --- | --- | --- |
|  |  |  | Low | High |  | Low | High |  | Low | High |  |
| Gender | Male | 60 | 36 | 24 | 1.000 | 41 | 18 | 0.175 | 43 | 17 | 0.573 |
|  | Female | 20 | 12 | 8 |  | 17 | 3 |  | 13 | 7 |  |
| Age | <60 | 40 | 23 | 17 | 0.648 | 30 | 10 | 0.747 | 29 | 11 | 0.626 |
|  | ≥60 | 40 | 25 | 15 |  | 28 | 11 |  | 27 | 13 |  |
| Differentiation | Low | 34 | 17 | 17 | 0.169 | 27 | 7 | 0.612 | 23 | 11 | 0.334 |
|  | Medium | 37 |  |  |  | 25 | 12 | 0.491 | 28 | 9 | 0.426 |
|  | High | 2 | 2 | 0 |  | 1 | 0 |  | 2 | 0 |  |
| Size | <4.5 cm | 41 | 26 | 15 | 0.835 | 29 | 11 | 0.805 | 28 | 13 | 0.351 |
|  | ≥4.5 cm | 36 | 22 | 14 |  | 27 | 9 |  | 28 | 8 |  |
| TNM stage | I-II | 55 | 34 | 21 | 0.314 | 38 | 16 | 0.439 | 39 | 16 | 0.903 |
|  | III-IV | 11 | 5 | 6 |  | 9 | 2 |  | 8 | 3 |  |
| Serum AFP | ≤20 ng/mL | 35 | 24 | 11 | 0.203 | 24 | 10 | 0.423 | 28 | 7 | 0.687 |
|  | >20 ng/mL | 16 | 8 | 8 |  | 13 | 3 |  | 12 | 4 |  |
| HBV infection | No | 22 | 14 | 8 | 0.790 | 16 | 5 | 0.818 | 18 | 4 | 0.473 |
|  | Yes | 30 | 18 | 12 |  | 22 | 8 |  | 22 | 8 |  |
| HCV infection | No | 30 | 19 | 11 | 0.756 | 24 | 6 | 0.282 | 24 | 6 | 0.539 |
|  | Yes | 22 | 13 | 9 |  | 14 | 7 |  | 16 | 6 |  |
| Survival | <6 years | 29 | 15 | 14 | 0.210 | 22 | 6 | 0.413 | 19 | 10 | 0.424 |
|  | ≥6 years | 50 | 33 | 17 |  | 35 | 15 |  | 37 | 13 |  |

**Supplementary Table 3. Multivariate analysis (Cox regression) of independent prognostic factors in patients with HCC.**

| **Prognostic factors** | **Harzard Ratio** | **95% CI** | **p-value** |
| --- | --- | --- | --- |
| *NTT* expression |  |  |  |
| Low | 0.399 | 0.153 to 1.037 | 0.059 |
| High | 1 |  |  |
| Gender |  |  |  |
| Male | 1.477 | 0.547 to 3.989 | 0.442 |
| Female | 1 |  |  |
| Age |  |  |  |
| <60 | 0.284 | 0.102 to 0.789 | 0.016 |
| ≥60 | 1 |  |  |
| Differentiation |  |  |  |
| Low | 3.757 | 1.513 to 9.325 | 0.004 |
| Medium/High | 1 |  |  |
| Tumor Size |  |  |  |
| <4.5 cm | 0.684 | 0.266 to 1.754 | 0.429 |
| ≥4.5 cm | 1 |  |  |
| Stage |  |  |  |
| I/II | 0.243 | 0.082 to 0.714 | 0.01 |
| III/IV | 1 |  |  |
